# 24-Hour Profiles of 11-Oxygenated C_19_ Steroids and Δ^5^-Steroid Sulfates during Oral and Continuous Subcutaneous Glucocorticoids in 21-Hydroxylase Deficiency

**DOI:** 10.3389/fendo.2021.751191

**Published:** 2021-11-16

**Authors:** Adina F. Turcu, Ashwini Mallappa, Aikaterini A. Nella, Xuan Chen, Lili Zhao, Aya T. Nanba, James Brian Byrd, Richard J. Auchus, Deborah P. Merke

**Affiliations:** ^1^ Division of Metabolism, Endocrinology and Diabetes, University of Michigan, Ann Arbor, MI, United States; ^2^ Pediatric Service, National Institutes of Health (NIH) Clinical Center, Bethesda, MD, United States; ^3^ Division of Pediatric Diabetes and Endocrinology, Baylor College of Medicine, Houston, TX, United States; ^4^ School of Public Health, University of Michigan, Ann Arbor, MI, United States; ^5^ Division of Cardiovascular Medicine, University of Michigan, Ann Arbor, MI, United States; ^6^ Department of Pharmacology, University of Michigan, Ann Arbor, MI, United States; ^7^ Eunice Kennedy Shriver National Institute of Child Health and Human Development, Bethesda, MD, United States

**Keywords:** 21-hydroxylase deficiency, congenital adrenal hyperplasia, 11-oxyandrogens, Circadian hormones, cortisol 24-hour profile, CAH

## Abstract

**Background:**

Optimal management of androgen excess in 21-hydroxylase deficiency (21OHD) remains challenging. 11-oxygenated-C_19_ steroids (11-oxyandrogens) have emerged as promising biomarkers of disease control, but data regarding their response to treatment are lacking.

**Objective:**

To compare the dynamic response of a broad set of steroids to both conventional oral glucocorticoids (OG) and circadian cortisol replacement *via* continuous subcutaneous hydrocortisone infusion (CSHI) in patients with 21OHD based on 24-hour serial sampling.

**Participants and Methods:**

We studied 8 adults (5 women), ages 19-43 years, with poorly controlled classic 21OHD who participated in a single-center open-label phase I–II study comparing OG with CSHI. We used mass spectrometry to measure 15 steroids (including 11-oxyandrogens and Δ^5^ steroid sulfates) in serum samples obtained every 2 h for 24 h after 3 months of stable OG, and 6 months into ongoing CSHI.

**Results:**

In response to OG therapy, androstenedione, testosterone (T), and their four 11-oxyandrogen metabolites:11β-hydroxyandrostenedione, 11-ketoandrostenedione, 11β-hydroxytestosterone and 11-ketotestosterone (11KT) demonstrated a delayed decline in serum concentrations, and they achieved a nadir between 0100-0300. Unlike DHEAS, which had little diurnal variation, pregnenolone sulfate (PregS) and 17-hydoxypregnenolone sulfate peaked in early morning and declined progressively throughout the day. CSHI dampened the early ACTH and androgen rise, allowing the ACTH-driven adrenal steroids to return closer to baseline before mid-day. 11KT concentrations displayed the most consistent difference between OG and CSHI across all time segments. While T was lowered by CSHI as compared with OG in women, T increased in men, suggesting an improvement of the testicular function in parallel with 21OHD control in men.

**Conclusion:**

11-oxyandrogens and PregS could serve as biomarkers of disease control in 21OHD. The development of normative data for these promising novel biomarkers must consider their diurnal variability.

## Introduction

Congenital adrenal hyperplasia (CAH) is a set of autosomal recessive defects in cortisol biosynthesis, and of these, 21-hydroxylase deficiency (21OHD) represents the most common form (1, 2). The molecular spectrum of 21OHD is wide, ranging from absent or minimal 21-hydroxylase activity, to fully compensated enzymatic defects (3). Conventionally, 21OHD forms associated with clinically overt adrenal insufficiency are collectively grouped into “classic” 21OHD, while cases with normal or near normal cortisol production are termed “non-classic” (3). The traditional biomarkers of 21OHD control are the androgen precursors 17α-hydroxyprogesterone (17OHP) and androstenedione (A4).

All forms of 21OHD are characterized by variable excess in adrenal androgen synthesis, which is driven by ACTH. Clinical manifestations of hyperandrogenism range from severe virilization, premature adrenarche or puberty, to hirsutism, acne, and infertility. Glucocorticoids have been the mainstay of treatment for patients with classic 21OHD, not only as replacement therapy for adrenal insufficiency, but also to lower ACTH and the resulting androgen surplus. With conventional oral glucocorticoid (OG) therapy, however, supra-physiological doses and nighttime administration are typically needed to reduce the early morning ACTH rise. Supra-physiologic glucocorticoid use contributes to higher rates of obesity, metabolic syndrome, and bone mass decline in adults with 21OHD (4, 5). Efforts towards optimizing the management of hyperandrogenism in patients with 21OHD while minimizing glucocorticoid daily dose include the development of modified-release OG (6) and continuous glucocorticoid delivery systems (7, 8), as well as a variety of non- glucocorticoid therapies (9). Continuous subcutaneous hydrocortisone infusion (CSHI) that approximates circadian cortisol secretion was previously shown to improve disease control in patients with 21OHD poorly controlled with OG therapy based on the traditional biomarkers 17OHP and A4 (7).

Additional challenges in the management of 21OHD derive from the uncertainties surrounding the optimal use of biomarkers to assess disease control (10, 11). The conventional steroid panel of patients with 21OHD has been expanded in the recent years, to include previously neglected androgens (12–14). In particular, a set of 11-oxygenated-C_19_ steroids (11-oxyandrogens), including 11β-hydroxyandrostenedione (11OHA4) and 11-ketotestosterone (11KT), have been shown to be elevated in patients with classic (12, 15) and non-classic 21OHD (16). 11-oxyandrogens were found to correlate with parameters of poor 21OHD control (11, 17). The dynamic responses of 11-oxyandrogens to glucocorticoid therapy have not been characterized, and this aspect will be key for clinical interpretation. Herein, we compared the 24-hour response of serum 11-oxyandrogens and conventional androgens to standard OG *vs.* CSHI in patients with classic 21OHD.

## Subjects and Methods

### Patients and Study Design

Eight adult patients (five women) with difficult to control classic 21OHD seen at the National Institutes of Health (NIH), Bethesda, Maryland were included in an open-label phase I–II study (NCT01859312) comparing CSHI with conventional OG, as previously described (7). The diagnosis of classic 21OHD was confirmed by hormonal and genetic analyses in all participants.

All patients were treated with stable OG doses for at least 3 months prior to study entry. For the CSHI, the total daily hydrocortisone dose was calculated based on the patient’s estimated cortisol clearance, which was conducted in each patient at baseline (7). Patients were admitted for frequent hormonal sampling (every 2 hours) on two separate occasions: at study entrance, while receiving conventional OG, and 6 months after initiation of CSHI. The OG equivalent dose (mg/m^2^/day) was calculated using a factor of 1 for hydrocortisone, 5 for prednisone and prednisolone, and 80 for dexamethasone (5).

### Hormonal Assays

We quantified 15 steroids by liquid chromatography-tandem mass spectrometry (LC-MS/MS), including: cortisol, 17OHP, 16α-hydroxyprogesterone (16OHP), 21-deoxycortisol (21dF), progesterone, A4, testosterone (T), 11β-hydroxyandrostenedione (11OHA4), 11-ketoandrostenedion (11KA4), 11β-hydroxytestosterone (11OHT), 11-ketotestosterone (11KT), pregnenolone sulfate (PregS), 17-hydoxypregnenolone sulfate (17OHPregS), dehydroepiandrosterone sulfate (DHEAS) and androst-5-ene-3β,17β-diol sulfate (AdiolS). Steroid extraction and quantitation were carried out as previously described (12, 13). Plasma ACTH was analyzed at the NIH Clinical Center, Bethesda, Maryland by chemiluminescent immunoassay on Siemens Immulite 200 XPi analyzer (7).

### Statistical Analyses

Logarithmic transformation was used to achieve normal data distribution where needed. The linear-up log-down trapezoidal rule was used to calculate the areas under the curve (AUC) for the 24 h periods studied, as well as for the morning (0500-1100h) and evening (1700-2300h) segments. Wilcoxon signed-rank test was used to compare AUCs between the two therapies. We assessed correlations between steroids and ACTH with the Spearman correlation test. Data analysis was conducted with R 3.6.2 (R Foundation for Statistical Computing). Two-tailed *p* values <0.05 were deemed statistically significant.

## Results

Of the eight participants, five were women, age 19–42 years, and three men, age 25–43 years. The median BMI was 40.1 kg/m^2^ (range: 27.7-54.1 kg/m^2^). While receiving standard OG therapy, the glucocorticoid equivalent doses ranged from 8.8 to 26.5 mg/m^2^/day.

**Figure 1:**
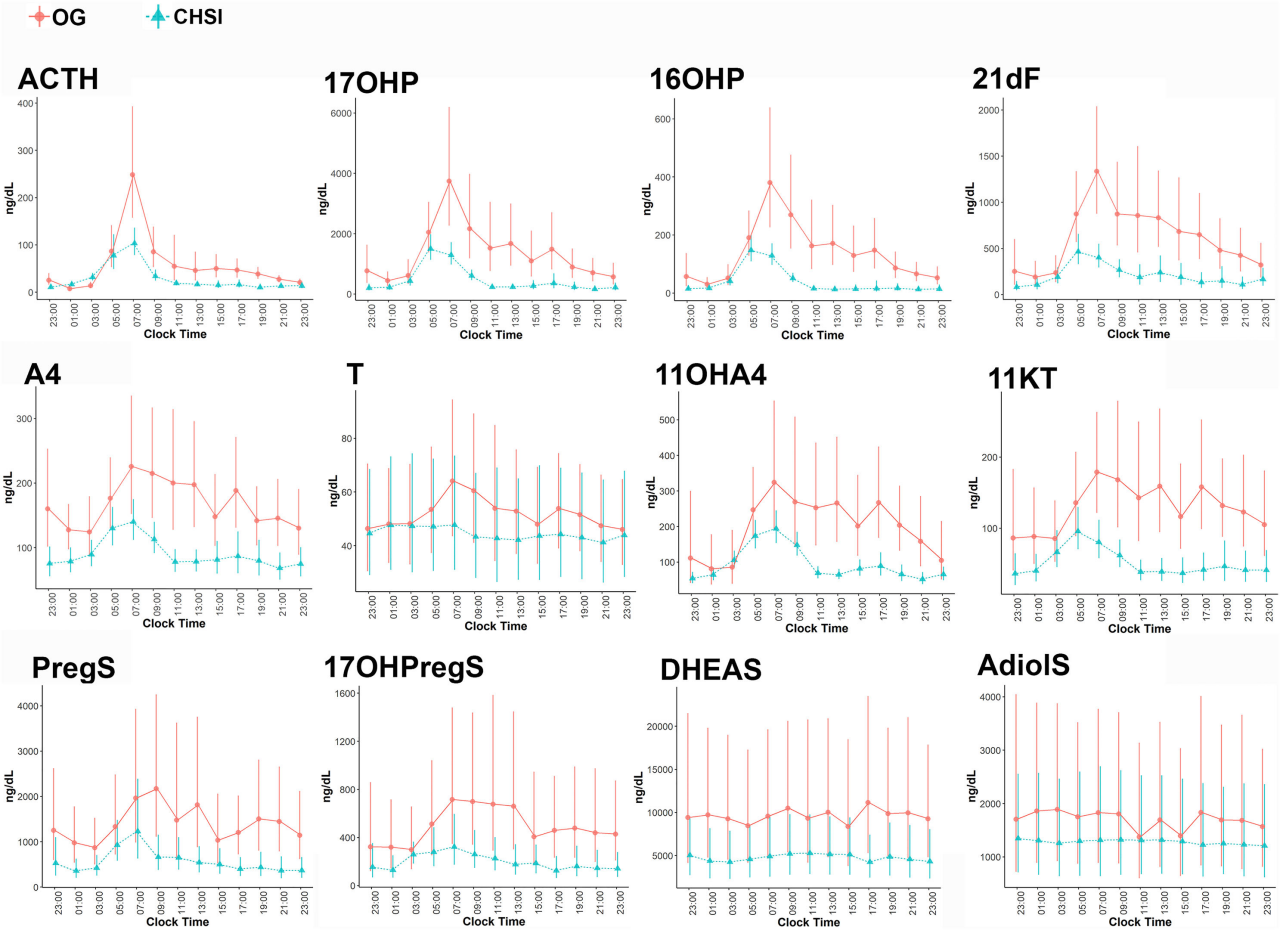
Serum concentrations of ACTH and steroids during conventional oral glucocorticoid therapy (OG, circles), and into 6 months of continuous subcutaneous hydrocortisone infusion (CSHI, triangles). Central data represent the geometrical means of all participants, and the vertical lines represent the standard errors of the geometrical means. 21dF, 21-deoxycortisol; 17OHProg, 17α-hydroxyprogesterone; 16OHProg, 16α-hydroxyprogesterone; A4, androstenedione; 11OHA4, 11β-hydroxyandrostenedione; 11KT, 11-ketotestosterone; T, testosterone PregS, pregnenolone sulfate; 17OHPregS, 17α-hydroxypregnenolone sulfate; DHEAS, dehydroepiandrosterone sulfate; AdiolS, Androstenediol-3-sulfate.

During conventional OG therapy, ACTH demonstrated a sharp peak around 0700h with a nadir 2300-0300h, which was mirrored by 17OHP, 16OHP and 21dF (**Figure 1**). A4, T, and their four 11-oxyandrogen metabolites demonstrated a delayed decline in serum concentrations, and they achieved a nadir between 0100-0300h (**Figure 1**). Not surprisingly, DHEAS and AdiolS displayed relatively flat circadian levels, with no significant differences between morning and nocturnal concentrations (**Figure 1** and **Table 1**); conversely, PregS and 17OHPregS peaked in early morning and declined progressively throughout the day.

**Table 1:** Comparison of steroid concentrations between 0700h and 0100h during oral glucocorticoid treatment.

Hormone (ng/dL)	0100 h	0700 h	*p* value
21dF	204.7 [71.7, 1079.4]	864.6 [530.6, 2885]	0.008
16OHProg	16.3 [14.7, 147.7]	252.8 [185, 1313.7]	0.008
17OHProg	243.5 [151.4, 1592.9]	3445.3 [2080.1, 11788.9]	0.008
T	58.4 [24.8, 91.7]	74 [32, 126.9]	0.023
A4	104.3 [66.1, 214.8]	189.3 [111.1, 459.7]	0.016
Prog	24.5 [7, 52.3]	138.5 [28.8, 204.1]	0.016
11OHT	24.2 [11.9, 52.1]	42.5 [32.5, 91]	0.039
11KT	83.7 [49.6, 197.2]	145.8 [125.8, 292.6]	0.008
11OHA4	97.4 [64.3, 507.5]	301.5 [122.4, 955.8]	0.039
11KA4	16.4 [12.3, 77.8]	46.7 [29.2, 133.3]	0.008
PregS	820.9 [661.8, 1490.1]	3104.8 [745.7, 4940]	0.039
17OHpregS	292.9 [220, 1715]	781.7 [347.7, 3138.5]	0.055
DHEAS	17974.4 [3627.5, 32826.8]	11740.4 [4455.3, 34320.6]	0.742
AdiolS	3919.3 [973.9, 9506.8]	3935.2 [1124.5, 7723.7]	1

Data are expressed as medians and interquartile ranges. Comparisons of baseline 0700h and 0100h steroid values were done with the Wilcoxon signed-rank test.

21dF, 21-deoxycortisol; 17OHProg, 17α-hydroxyprogesterone; 16OHP, 16α-hydroxyprogesterone; A4, androstenedione; 11OHA4, 11β-hydroxyandrostenedione; 11OHT, 11β-hydroxytestosterone; 11KA4, 11-ketoandrostenedione; 11KT, 11-ketotestosterone; T, testosterone PregS, pregnenolone sulfate; 17OHPregS, 17α-hydroxypregnenolone sulfate; DHEAS, dehydroepiandrosterone sulfate; AdiolS, Androstenediol-3-sulfate.

**Figure 2:**
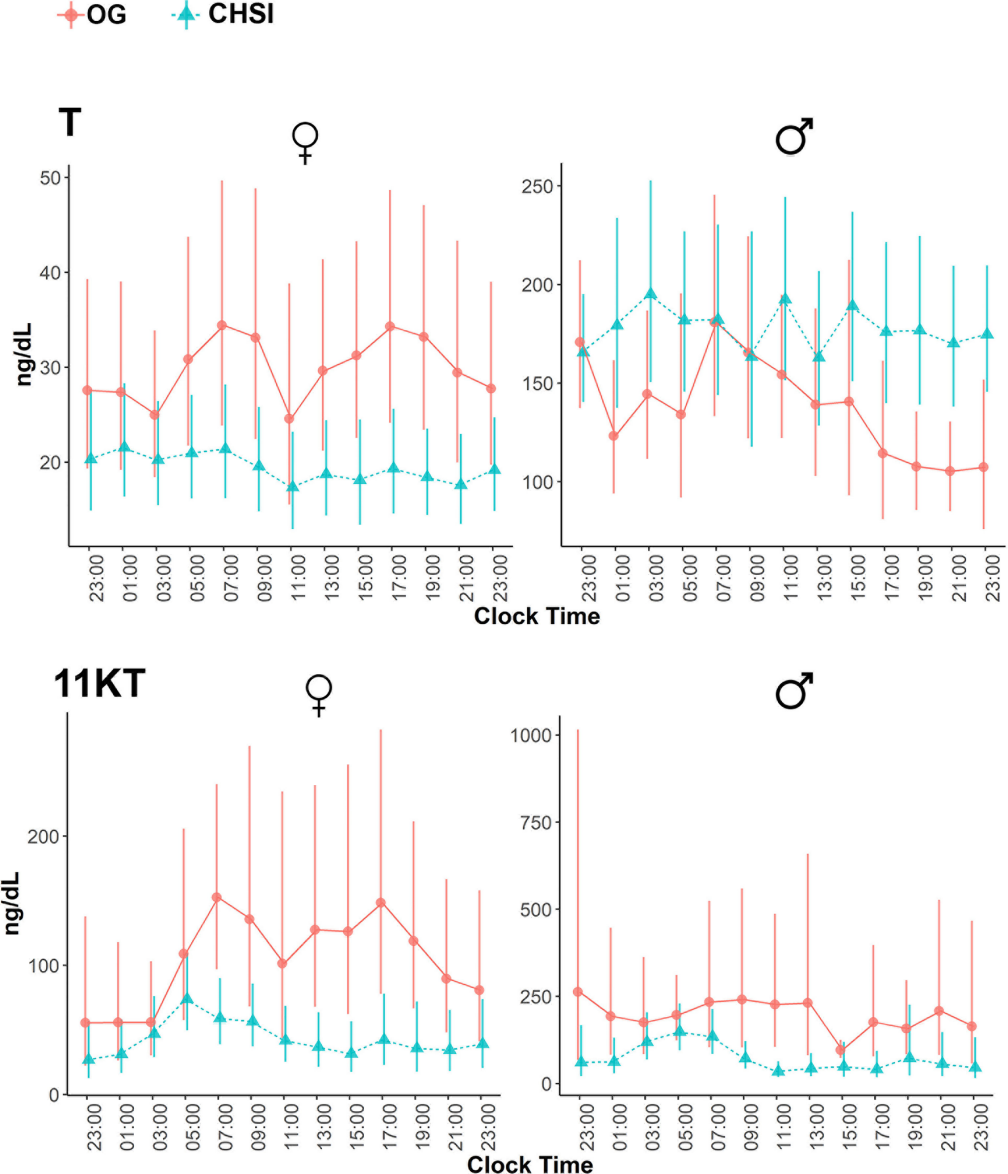
Serum concentrations of testosterone (T) and 11-ketotestosterone (11KT) during conventional oral glucocorticoid therapy (OG, circles), and into 6 months of continuous subcutaneous hydrocortisone infusion (CSHI, triangles), in women (N=5) and men (N=3). Central data represent the geometrical means, and the vertical lines represent the standard errors of the geometrical means.

Overall, most steroid concentrations were lower during CSHI as compared with OG therapy (**Figure 1** and **Table 2**). Following a more subtle morning ACTH peak during CSHI, steroids with diurnal fluctuations returned to baseline levels around 1100 (**Figure 1**) and remained stable throughout the rest of the day. Notably, while T was lowered by CSHI as compared with OG in women, T increased in men, suggesting an improvement of the testicular function in parallel with 21OHD control in men (**Figure 2**).

**Table 2:** Comparison of steroid concentrations during conventional oral glucocorticoid therapy (OG) *vs.* continuous subcutaneous hydrocortisone infusion (CSHI).

Hormone	Time segment	OG AUC (ng/dL x h)	CSHI AUC (ng/dL x h)	*p* value
**21dF**	24 h	12582.6 [7762.4, 36130.4]	4513.6 [2944.8, 17916.7]	0.023
	0500 – 1100 h	4387.3 [2862.4, 13169.3]	1492.6 [1005.3, 5058.6]	0.039
	1700 – 2300 h	2341.1 [1522.1, 5686.6]	839.2 [546.7, 2778.6]	0.109
**16OHProg**	24 h	3077.2 [1554.8, 12975.3]	1568.5 [545.2, 2378.7]	0.055
	0500 – 1100 h	1269.5 [749.2, 5329.3]	523.3 [294.5, 1036.9]	0.055
	1700 – 2300 h	436.2 [176.2, 1339.9]	113.8 [27.3, 381.4]	0.016
**17OHProg**	24 h	40649.6 [18989.8, 112674.8]	12074 [6734.9, 18718.8]	0.023
	0500 – 1100 h	16184.4 [8497.6, 46213.2]	5344.2 [3842.7, 7751.8]	0.023
	1700 – 2300 h	5904.4 [2279.9, 19004.2]	1177.7 [442.5, 2846.6]	0.016
**T**	24 h	1416.5 [725.8, 2379.3]	735 [562, 3244.8]	0.641
	0500 – 1100 h	413 [154.3, 693.2]	190.5 [142.8, 779.6]	0.641
	1700 – 2300 h	349.1 [187, 533]	177.8 [122.6, 831]	0.641
**A4**	24 h	3899 [2387.4, 7308]	2787 [1276.5, 3027.5]	0.023
	0500 – 1100 h	970.9 [717.7, 2202.5]	825.7 [496.7, 929.5]	0.078
	1700 – 2300 h	841.6 [572.5, 1693.4]	542.5 [195.9, 655.3]	0.039
**Prog**	24 h	2055.5 [398.1, 10706.6]	353.7 [94.7, 12189.2]	1
	0500 – 1100 h	611.7 [172.5, 2864.6]	86.1 [39.1, 2827.1]	0.844
	1700 – 2300 h	275.6 [61.4, 1184.3]	110.1 [18.7, 884.7]	0.461
**11OHT**	24 h	793.7 [372.8, 1343.5]	361.3 [171, 476.9]	0.023
	0500 – 1100 h	207.4 [131.6, 448.5]	188.2 [52.9, 211.7]	0.109
	1700 – 2300 h	189.6 [67, 334.2]	71.8 [32.9, 82.7]	0.023
**11KT**	24 h	2768.3 [2434.8, 6638]	1519.7 [989.4, 2327.9]	0.008
	0500 – 1100 h	758.4 [592, 1875.1]	503.1 [363.3, 612.8]	0.016
	1700 – 2300 h	774.5 [533.1, 1543.3]	269.8 [147.1, 646]	0.008
**11OHA4**	24 h	5439.8 [1875.6, 15742.4]	2794 [1819.4, 3864.6]	0.109
	0500 – 1100 h	1505 [597.3, 5155.2]	1230.5 [719.2, 1314.9]	0.25
	1700 – 2300 h	1266.4 [461.3, 3094.5]	322.1 [253.8, 740.7]	0.016
**11KA4**	24 h	856.9 [453.5, 2971.7]	595.3 [404.5, 675.2]	0.055
	0500 – 1100 h	261.3 [150.1, 858.6]	192 [161, 272.8]	0.078
	1700 – 2300 h	190.6 [92.6, 766.7]	102.1 [71.1, 120.5]	0.148
**PregS**	24 h	52529.3 [16205.8, 81068.3]	27883.1 [6585, 30451.4]	0.023
	0500 – 1100 h	15023.3 [4697.4, 27020.5]	9002.2 [2464, 12042.7]	0.078
	1700 – 2300 h	11567.1 [3900.8, 16714]	5011.3 [701.5, 5474.3]	0.008
**17OHPregS**	24 h	13783.5 [7435.8, 52818.6]	8054.5 [4519.1, 13381.3]	0.016
	0500 – 1100 h	4260.6 [1401.1, 17786.5]	2548.3 [1447.6, 4540.2]	0.023
	1700 – 2300 h	3148.5 [1428.9, 11432.8]	1739.8 [752.7, 2173]	0.008
**DHEAS**	24 h	278225.1 [110016.9, 921487]	177721.3 [53305.4, 425090.4]	0.008
	0500 – 1100 h	56092.5 [29166.4, 210158.1]	47525.4 [12323.5, 117190.3]	0.039
	1700 – 2300 h	62665.7 [27821.9, 263578.4]	43929 [14807.3, 81494.3]	0.008
**AdiolS**	24 h	89933.2 [25443.2, 181370.8]	62435.3 [13767.9, 129204.2]	0.039
	0500 – 1100 h	16444 [6832.4, 43720.4]	16332.5 [3043.2, 37080.1]	0.195
	1700 – 2300 h	19499.9 [5533.1, 44425.2]	10673.3 [3903.9, 29584.6]	0.055
**ACTH**	24 h	161.5 [106.0, 438.5]	71.0 [59.1, 109.6]	0.016
	0500 – 1100 h	88.3 [54.3, 219.9]	38.0 [27.1, 47.2]	0.016
	1700 – 2300 h	18.1 [15.4, 28.1]	7.7 [5.6, 20.5]	0.023

Data are expressed as medians and interquartile ranges of areas under the curves (AUC).

21dF, 21-deoxycortisol; 17OHProg, 17α-hydroxyprogesterone; 16OHP, 16α-hydroxyprogesterone; A4, androstenedione; 11OHA4, 11β-hydroxyandrostenedione; 11OHT, 11β-hydroxytestosterone; 11KA4, 11-ketoandrostenedione; 11KT, 11-ketotestosterone; T, testosterone PregS, pregnenolone sulfate; 17OHPregS, 17α-hydroxypregnenolone sulfate; DHEAS, dehydroepiandrosterone sulfate; AdiolS, Androstenediol-3-sulfate.

Of the steroids measured, 17OHP and 16OHP correlated best with ACTH (r=0.81 and 0.77, respectively, *p*<0.0001), followed by A4 (r=0.68), PregS (r=0.62), and 11KT (r=0.61, *p*<0.0001 for all). A4 and its 11-oxygenated derivatives correlated tightly among each other, as well as with 11OHT and 11KT (**Table 3**). Conversely, 11KT correlated directly with T only in women, while in men, 11KT and T displayed an inverse correlation, similar to our previous studies (12, 17), supporting their origin from independent sources in well-controlled men.

**Table 3:** Correlations between steroids and ACTH.

		ACTH	21dF	16OHP	17OHP	Prog	T	11OHT	11KT	A4	11OHA4	11KA4	PregS	17OHPregS	DHEAS	AdiolS
**ACTH**	r	1.00	0.56	0.78	0.82	0.29	0.13	0.47	0.61	0.68	0.48	0.42	0.61	0.44	0.21	0.15
	*p*		<.001	<.001	<.001	<.001	0.073	<.001	<.001	<.001	<.001	<.001	<.001	<.001	0.003	0.038
**21dF**	r	0.56	1.00	0.87	0.76	0.62	0.47	0.67	0.84	0.64	0.74	0.74	0.63	0.77	0.59	0.34
	*p*	<.001		<.001	<.001	<.001	<.001	<.001	<.001	<.001	<.001	<.001	<.001	<.001	<.001	<.001
**16OHP**	r	0.78	0.87	1.00	0.93	0.58	0.35	0.65	0.82	0.74	0.71	0.68	0.66	0.64	0.40	0.23
	*p*	<.001	<.001		<.001	<.001	<.001	<.001	<.001	<.001	<.001	<.001	<.001	<.001	<.001	0.009
**17OHP**	r	0.82	0.76	0.93	1.00	0.38	0.35	0.61	0.81	0.86	0.70	0.63	0.74	0.56	0.39	0.30
	*p*	<.001	<.001	<.001		<.001	<.001	<.001	<.001	<.001	<.001	<.0001	<.01	<.001	<.001	<.001
**Prog**	r	0.29	0.62	0.58	0.38	1.00	0.20	0.32	0.40	0.19	0.37	0.38	0.18	0.46	0.23	-0.02
	*p*	<.0001	<.0001	<.0001	<.0001		0.004	<.0001	<.0001	0.0072	<.0001	<.0001	0.0099	<.0001	0.0012	0.7535
**T**	r	0.13	0.47	0.35	0.35	0.20	1.00	0.23	0.41	0.29	0.41	0.41	0.22	0.26	0.18	0.14
	*p*	0.07	<.001	<.001	<.001	0.004		0.001	<.001	<.0001	<.001	<.001	0.002	0.001	0.0082	0.0432
**11OHT**	r	0.47	0.67	0.65	0.61	0.32	0.23	1.00	0.77	0.72	0.81	0.78	0.71	0.62	0.73	0.60
	*p*	<.001	<.001	<.001	<.001	<.001	0.001		<.001	<.001	<.001	<.001	<.001	<.001	<.001	<.001
**11KT**	r	0.61	0.84	0.82	0.81	0.40	0.41	0.77	1.00	0.82	0.72	0.68	0.84	0.83	0.70	0.58
	*p*	<.001	<.001	<.001	<.001	<.001	<.001	<.001		<.001	<.001	<.001	<.001	<.001	<.001	<.001
**A4**	r	0.68	0.64	0.74	0.86	0.19	0.29	0.72	0.82	1.00	0.76	0.65	0.85	0.60	0.64	0.60
	*p*	<.001	<.001	<.001	<.001	0.007	<.001	<.001	<.001		<.001	<.001	<.001	<.001	<.001	<.001
**11OHA4**	r	0.48	0.74	0.71	0.70	0.37	0.41	0.81	0.72	0.76	1.00	0.87	0.60	0.55	0.68	0.54
	*p*	<.001	<.001	<.001	<.001	<.001	<.001	<.001	<.001	<.001		<.001	<.001	<.001	<.001	<.001
**11KA4**	r	0.42	0.74	0.68	0.63	0.38	0.41	0.78	0.68	0.65	0.87	1.00	0.55	0.53	0.61	0.44
	*p*	<.001	<.001	<.001	<.001	<.001	<.001	<.001	<.001	<.001	<.001		<.001	<.001	<.001	<.001
**PregS**	r	0.61	0.63	0.66	0.74	0.18	0.22	0.71	0.84	0.85	0.60	0.55	1.00	0.81	0.76	0.75
	*p*	<.0001	<.001	<.001	<.001	0.01	0.002	<.001	<.001	<.001	<.001	<.001		<.001	<.001	<.001
**17OHPregS**	r	0.44	0.77	0.64	0.56	0.46	0.26	0.62	0.83	0.60	0.55	0.53	0.81	1.00	0.80	0.65
	*p*	<.001	<.001	<.001	<.001	<.001	0.001	<.001	<.001	<.001	<.001	<.001	<.001		<.001	<.001
**DHEAS**	r	0.21	0.59	0.40	0.39	0.23	0.18	0.73	0.70	0.64	0.68	0.61	0.76	0.80	1.00	0.90
	*p*	0.003	<.001	<.001	<.001	0.001	0.008	<.001	<.001	<.001	<.001	<.001	<.001	<.001		<.001
**AdiolS**	r	0.15	0.34	0.23	0.30	-0.02	0.14	0.60	0.58	0.60	0.54	0.44	0.75	0.65	0.90	1.00
	*p*	0.038	<.001	0.01	<.001	0.75	0.04	<.001	<.001	<.001	<.001	<.001	<.001	<.001	<.001	

21dF, 21-deoxycortisol; 17OHP, 17a-hydroxyprogesterone; 16OHP, 16a-hydroxyprogesterone; A4, androstenedione; 11OHA4, 11b-hydroxyandrostenedione; 11OHT, 11b-hydroxytestosterone; 11KA4, 11-ketoandrostenedione; 11KT, 11-ketotestosterone; T, testosterone PregS, pregnenolone sulfate; 17OHPregS, 17a-hydroxypregnenolone sulfate; DHEAS, dehydroepiandrosterone sulfate; AdiolS, Androstenediol-3-sulfate.

## Discussion

The circadian rhythmicity of cortisol is regulated *via* ACTH (18) as dictated by the hypothalamic suprachiasmatic nucleus (19). Physiologic cortisol synthesis has a characteristic circadian pattern, with an early morning peak and a late-night nadir (20–22). In patients with classic 21OHD, the early morning rise in ACTH is further amplified by the reduced negative feedback from cortisol. Like cortisol, 11-oxyandrogens are synthesized *via* the adrenal cortical enzyme 11β-hydroxylase (CYP11B1), which is regulated by ACTH (23). Although 11KT is produced primarily from adrenal-derived 11OHA4 *via* peripheral metabolism, we found that 11KT still correlates highly with ACTH, in agreement with an earlier study (17). In healthy individuals, 11-oxyandrogens display morning peaks and nocturnal nadirs, similar to cortisol (22). In a recent study of patients with classic 21OHD, salivary 11OHA4 and 11KT were highest in the morning, and declined by evening (24). Similarly, based on 24-hour serial sampling, we here show that in patients with 21OHD treated with conventional OG therapy, all four 11-oxyandrogens peak in the morning, following the sharp rise of ACTH; the 11-oxyandrogens decline modestly throughout the day, to eventually reach nadirs between 0100-0300h, demonstrating a diurnal rhythm. Conversely, during CSHI, the serum concentrations of 11-oxyandrogens return to baseline levels after 1100h, reflecting the improved disease control achieved with circadian cortisol replacement.

11KT is a potent androgen, with bioactivity similar to that of T (25–27). Furthermore, similar to the conversion of T to dihydrotestosterone (DHT) in target tissues, 11KT is a substrate for 5α-reductases (28). 11KT and its precursors are associated with clinical indicators of poor 21OHD control, including large adrenal volume, presence of testicular adrenal rest tumor in males, and irregular menses in females (17). As such, normalization of 11KT and its precursors is expected to be an important clinical goal in patients with 21OHD. In contrast with T, which also derives from the gonads, 11KT reflects the adrenal component of androgen production alone.11KT is particularly useful in men with 21OHD, who might have normal serum T concentration despite poor 21OHD control, suppressed gonadotropins, and infertility. In such men, the deceivingly normal serum T concentration represents a disproportionately high adrenal contribution. Repression of the early rise in ACTH with CSHI led to an increase in T in men and a decline of T in women, while 11KT decreased in both sexes. This divergence in T and 11KT origin in men following ACTH lowering is also reflected by the inverse correlation of the two steroids. In contrast, T and 11KT correlated positively in women with 21OHD, as both steroids are produced primarily by the adrenal glands in these women.

Although quantitatively abundant, DHEA and DHEAS have virtually no androgenic activity (25). Moreover, DHEAS is a poor biomarker of 21OHD control, which can be paradoxically low in poorly controlled classic 21OHD patients (12, 29). This enigma could be partly explained by the relative shift in sulfotransferase type 2A (SULT2A1) substrates, as suggested by the higher PregS serum concentrations in patients with 21OHD *vs.* age and BMI-matched controls (12). We have previously shown both *in vivo* and *in vitro*, that in contrast with DHEAS and AdiolS, PregS responds acutely to ACTH stimulation (30). PregS has also been found to be associated with clinical surrogates of poor 21OHD control (17). Thus, PregS could be more relevant than DHEAS in the clinical evaluation of patients with 21OHD.

Practices vary regarding the laboratory evaluation of patients with 21OHD, and 24-hour sampling is only done in a research setting. Monitoring treatment by consistently timed hormone measurements relative to the time of glucocorticoid administration is recommended (31), as glucocorticoid acutely suppress adrenal steroid production. Although 17OHP and A4 show good correlation, we previously found that 17% of laboratory evaluations done in the early morning resulted in discrepant findings between these two traditional biomarkers (11). 11-oxyandrogens are the major androgens and androgen precursors found in classic and nonclassic 21OHD (12, 16) and likely provide more direct evidence of adrenal androgen excess. However, their use in the medical management and monitoring of 21OHD has yet to be determined. Our study of 24-hour steroid profiles of these newly defined biomarkers in addition to the traditional biomarkers provides insight into the 24-hour rhythms of adrenal androgens in patients with classic 21OHD. Our findings suggest that 11-oxyandrogens remain elevated for a longer period of time than 17OHP in patients with poor disease control and demonstrate a circadian rhythm.

In summary, this is the first study to show 24-hour profiles of 11-oxyandrogens and related Δ^5^- and Δ^4^-steroids in patients with 21OHD. We directly compared the dynamic responses of 21OHD biomarkers, including adrenal 11-oxyandrogens and Δ^5^ steroid sulfates, to both conventional OG therapy and CSHI. We found that the high amplitude ACTH morning rise that occurs in patients treated with OG leads to sharp elevations of several adrenal steroids, particularly those immediately preceding the enzymatic blockade. Following such marked ACTH stimulation, 11-oxyandrogen elevations decline rather slowly throughout the day. This finding has clinical implications, especially since 11KT has androgenic activity equivalent to T. Conversely, mimicking physiologic cortisol concentrations *via* CSHI dampens the early ACTH rise, allowing ACTH-driven adrenal steroids to return closer to baseline before mid-day. In addition to ACTH, these dynamic steroid excursions are also impacted by protein binding and metabolism, which remain understudied. Additionally, the small number of patients and lack of nonclassic 21OHD and healthy individuals as comparators limit the extrapolation of the absolute values for broader use. Nevertheless, our data reinforce the potential utility of 11-oxyandrogens as biomarkers of disease control. The development of normative data and the establishment of 21OHD monitoring recommendations for the promising biomarkers – both 11-oxyandrogens and PregS – needs to consider their diurnal variability.

## Data Availability Statement

The raw data supporting the conclusions of this article will be made available by the authors, without undue reservation.

## Ethics Statement

The studies involving human participants were reviewed and approved by National Institutes of Health Institutional Review Board. The patients/participants provided their written informed consent to participate in this study.

## Author Contributions

AM, AAN, and DM were investigators in the clinical study. AT proposed the current study. ATN performed mass spectrometry steroid assays. XC, LZ, JB, and AT analyzed the data. AT, DM, and RA interpreted the data. AT drafted the manuscript. All authors contributed to the article and approved the submitted version.

## Funding

This work was supported, in part, by grants 1K08DK109116 (to AT), K23HL128909 (to JB), and R01GM086596 (to RA) from the National Institutes of Health, by Michigan Institute for Clinical and Health Research (MICHR) Translational Science Award/U046500 to AT and, in part, by the Intramural Research Program of the National Institutes of Health.

## Conflict of Interest

DM received unrelated research funds from Diurnal Limited through the National Institutes of Health Cooperative Research and Development Agreement. RA received unrelated research funds from Spruce Biosciences and Neurocrine Biosciences.

The remaining authors declare that the research was conducted in the absence of any commercial or financial relationships that could be construed as a potential conflict of interest.

## Publisher’s Note

All claims expressed in this article are solely those of the authors and do not necessarily represent those of their affiliated organizations, or those of the publisher, the editors and the reviewers. Any product that may be evaluated in this article, or claim that may be made by its manufacturer, is not guaranteed or endorsed by the publisher.
